# *In-situ* Formation of Amorphous Co-Al-P Layer on CoAl Layered Double Hydroxide Nanoarray as Neutral Electrocatalysts for Hydrogen Evolution Reaction

**DOI:** 10.3389/fchem.2020.552795

**Published:** 2020-10-22

**Authors:** Wanqing Teng, Zhaomei Sun, Junfeng Xie, Ziqiang Wang, Xiangjiang Zheng, Bo Tang

**Affiliations:** ^1^Shandong Provincial Key Laboratory of Detection Technology for Tumor Markers, School of Chemistry and Chemical Engineering, Linyi University, Linyi, China; ^2^Key Laboratory of Molecular and Nano Probes, Ministry of Education, Collaborative Innovation Center of Functionalized Probes for Chemical Imaging in Universities of Shandong, College of Chemistry, Chemical Engineering and Materials Science, Shandong Normal University, Jinan, China; ^3^College of Chemical Engineering, Zhejiang University of Technology, Hangzhou, China

**Keywords:** hydrogen evolution reaction, electrocatalysts, neutral pH, phosphide, nanoarray

## Abstract

Exploration of high-efficiency and inexpensive electrode catalysts is of vital importance for the hydrogen evolution reaction (HER). In this research, an amorphous Co-Al-P layer was constructed on the surface of CoAl layered double hydroxide (CoAl-LDH) via an *in-situ* wet phosphidation strategy. The core-shell CoAl-LDH@Co-Al-P on Ti mesh (CoAl-LDH@Co-Al-P/TM) as an active HER electrocatalyst demands an overpotential of 150 mV to achieve a current density of 10 mA cm^−2^ at neutral pH. Moreover, CoAl-LDH@Co-Al-P/TM also exhibits good electrochemical stability and a superior Faradic efficiency of nearly 100%.

## Introduction

Recently, the ever-increasing energy crisis and environmental pollution have become serious global concerns caused by the excessive consumption of fossil fuels. There is an urgent demand for clean and sustainable energy resources to replace the traditional fossil fuels (Chow et al., 2003; Chu and Majumdar, 2012; Xie and Xie, 2015; Huang L. et al., 2019). Hydrogen has been regarded as one of the most ideal candidates because of its high energy density and zero-carbon emissions (Sun et al., 2018; Chi et al., 2019; Fei et al., 2020; Zhu et al., 2020), and abundant efforts have been devoted to exploring appropriate strategies for hydrogen production. Up to date, it is well-established that water electrolysis is a promising and environmentally friendly approach to produce highly pure hydrogen (Wang et al., 2018a; Hu et al., 2019; Chen et al., 2020a; Xue et al., 2020). Nowadays, it is highly attractive to develop and construct active and stable electrode catalysts which make the hydrogen evolution reaction (HER) more energy efficient. As we know, the most efficient electrode catalysts for the HER are Pt-based noble metal materials, but their low abundance and high cost substantially hamper their commercial applications in water electrolysis (Zhou et al., 2016; Hu et al., 2017; Chen et al., 2019, 2020b; Huang T. et al., 2019). Therefore, the exploration of low-cost, effective, and earth-abundant electrocatalysts is crucial for the efficient and large scale production of hydrogen (Wang et al., 2016). Despite the significant progress of electrochemical hydrogen generation in acidic/alkaline conditions, the harsh conditions of electrolytes would cause severe corrosion issues and the activities of most electrocatalysts would decline over successive tests. In fact, neutral electrolytes can tackle the problems related to harsh acidic/alkaline corrosion. Unfortunately, catalytic performance under a neutral environment is far less than that in acidic/alkaline media. Consequently, improving the HER performance of electrocatalysts at neutral solutions is highly imperative.

Co-based compounds, such as sulfides (Faber et al., 2014; Zhang H. et al., 2015; Li N. et al., 2017), oxides (Li R. et al., 2017; Ling et al., 2017), nitrides (Wang Y. et al., 2017; Chen et al., 2018a), and phosphides (Liu et al., 2014; Tian et al., 2014; Xu et al., 2017; Zhang C. et al., 2017) have attracted extensive attention and exhibited comparable HER activities to noble metal catalysts. Among the various Co-based HER catalysts, phosphides hold high potential for future energy owing to their metal-like properties and high electrical conductivity. However, toxic gases are produced in the typical synthesis process which seriously impedes the large-scale application of phosphide electrocatalysts.

CoAl layered double hydroxides (CoAl-LDH) have attracted widespread interest in supercapacitor electrodes (Zhang A. et al., 2015; Zai et al., 2017), magnetic materials (Wang et al., 2011), and catalysts (Chen et al., 2013) due to their electrochemical characteristics. However, CoAl-LDHs have rarely been studied for HER catalysts owing to the re-stacking and relatively poor conductivity. It has been widely accepted that 3-dimensional (3D) nanoarray directly grown on conductive supports can improve the mass transfer efficiency, increase the active sites, and lessen the interfacial resistance. Moreover, recent work indicates that the conductivity can also be enhanced by forming a Co-Al LDH-carbon nanotube (CNT) composite (Yu et al., 2014) or coating them with platinum films (Cheng et al., 2013). However, creating a striking improvement in the HER performance is still an ongoing challenge that has many opportunities.

Inspired by the mentioned analysis, we constructed an amorphous Co-Al-P layer on CoAl-LDH nanosheets via an *in-situ* wet phosphidation strategy. Firstly, CoAl-LDH nanosheets were grown on the surface of 3D conductive Ti mesh (CoAl-LDH/TM) through the hydrothermal approach, followed by an *in-situ* partial conversion of CoAl-LDH into amorphous Co-Al-P via applying a bias into a specific solution. As expected, when used as a catalyst for the neutral electrochemical water reduction process, the resulting core-shell CoAl-LDH@Co-Al-P nanosheet array on Ti mesh (CoAl-LDH@Co-Al-P/TM) showed excellent catalytic activity in 1 M phosphate buffer solution (PBS, pH = 7). To attain the catalytic current density of 10 mA cm^−2^, CoAl-LDH@Co-Al-P/TM required an overpotential of 150 mV at a neutral pH. Remarkably, the CoAl-LDH@Co-Al-P/TM catalyst also offered good electrochemical stability and achieved an outstanding Faradic efficiency of nearly 100% under neutral conditions.

## Results and Discussion

**Figure 1:**
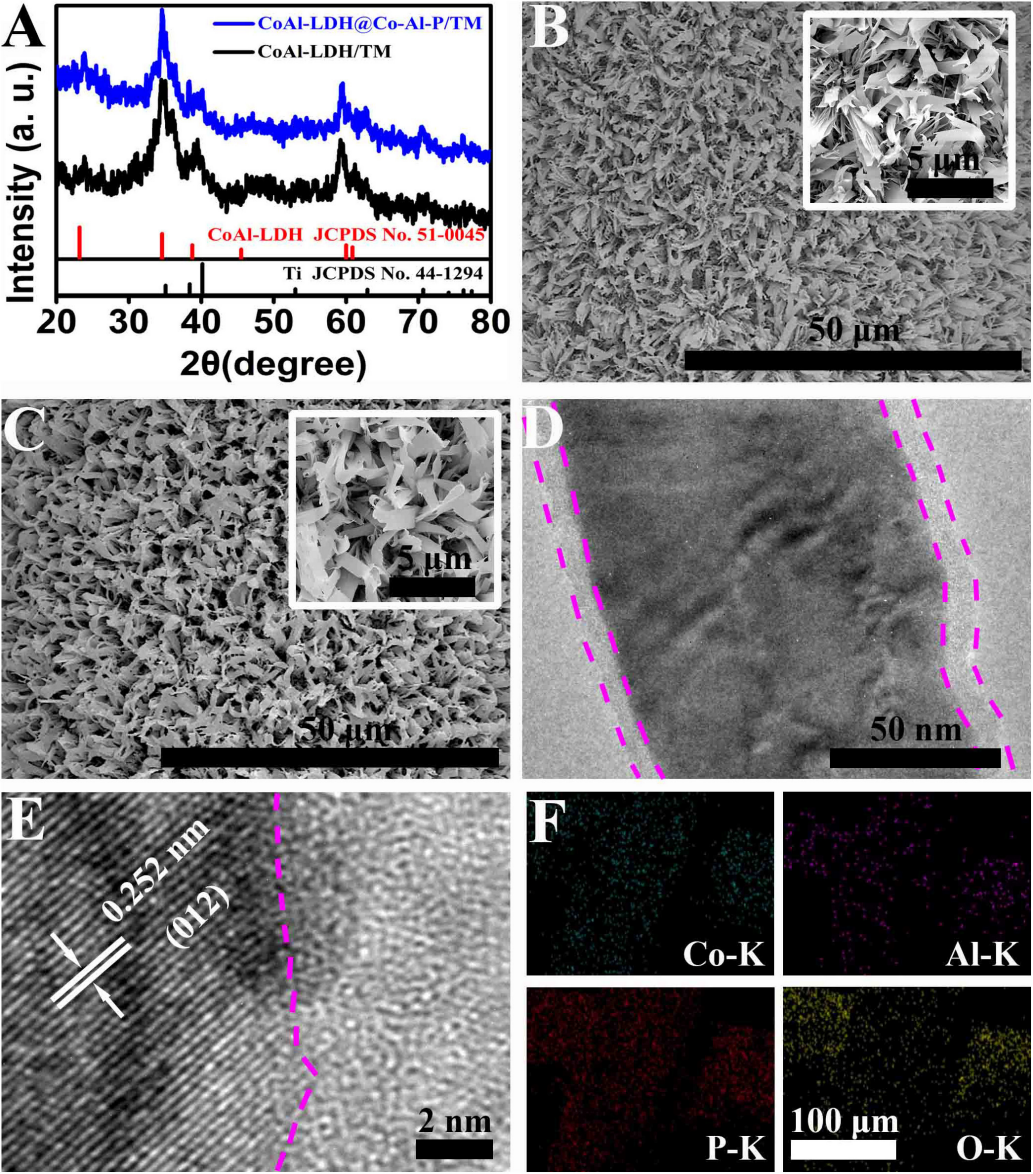
**(A)** XRD patterns of CoAl-LDH/TM and CoAl-LDH@Co-Al-P/TM. Low- and high-magnification SEM images for **(B)** CoAl-LDH/TM and **(C)** CoAl-LDH@Co-Al-P/TM. **(D)** TEM and **(E)** HRTEM images of CoAl-LDH@Co-Al-P. **(F)** EDX elemental mapping images of Co, Al, P, and O for CoAl-LDH@Co-Al-P, respectively.

The structural information of CoAl-LDH/TM and CoAl-LDH@Co-Al-P/TM were obtained from x-ray diffraction (XRD) patterns. As illustrated in Figure 1A, CoAl-LDH/TM exhibits diffraction peaks at around 23.8°, 34.6°, 39.4°, and 59.5° matched well with the (006), (012), (015), and (110) facets of CoAl-LDH (JCPDS card no. 51-0045), respectively, and other peaks belonging to Ti mesh (JCPDS card no. 44-1294). Note that, no new characteristic peaks were observed in the phosphide product except those of CoAl-LDH and Ti mesh, which meant that the generation of an amorphous phase occurred on the surface of CoAl-LDH. The scanning electron microscopy (SEM) images of CoAl-LDH/TM displayed that the surface of Ti mesh was densely covered by the CoAl-LDH nanosheet array (Figure 1B). Notably, the surface of CoAl-LDH@Co-Al-P/TM became a little rough but still retained the nanoarray feature after the *in-situ* transformation (Figure 1C). The results apparently show that CoAl-LDH@Co-Al-P/TM has a larger surface area than CoAl-LDH/TM, which are favorable for the enhancement of the HER activity. The transmission electron microscope (TEM, Figure 1D) image indicated the core-shell character of CoAl-LDH@Co-Al-P. The high-resolution transmission electron microscopy (HRTEM) image showed that the well-resolved lattice fringes with an interplanar distance of 0.252 nm were indexed to the (012) crystal plane of CoAl-LDH in the inner part and no crystal lattices existed in the shell (Figure 1E). Combined with the XRD results, we can conclude the formation of an amorphous Co-Al-P layer on the surface of CoAl-LDH via *in-situ* conversion. The energy-dispersive x-ray (EDX, Supplementary Figure 1A) spectrum confirmed the existence of the Co, Al, P, and O elements. EDX elemental mapping images (Supplementary Figure 1B and Figure 1F) demonstrated that the Co, Al, P, and O elements were homogenously distributed throughout the entire CoAl-LDH@Co-Al-P nanoarray. All the results clearly reveal the successful generation of a core-shell CoAl-LDH@Co-Al-P nanosheet array on Ti mesh.

**Figure 2:**
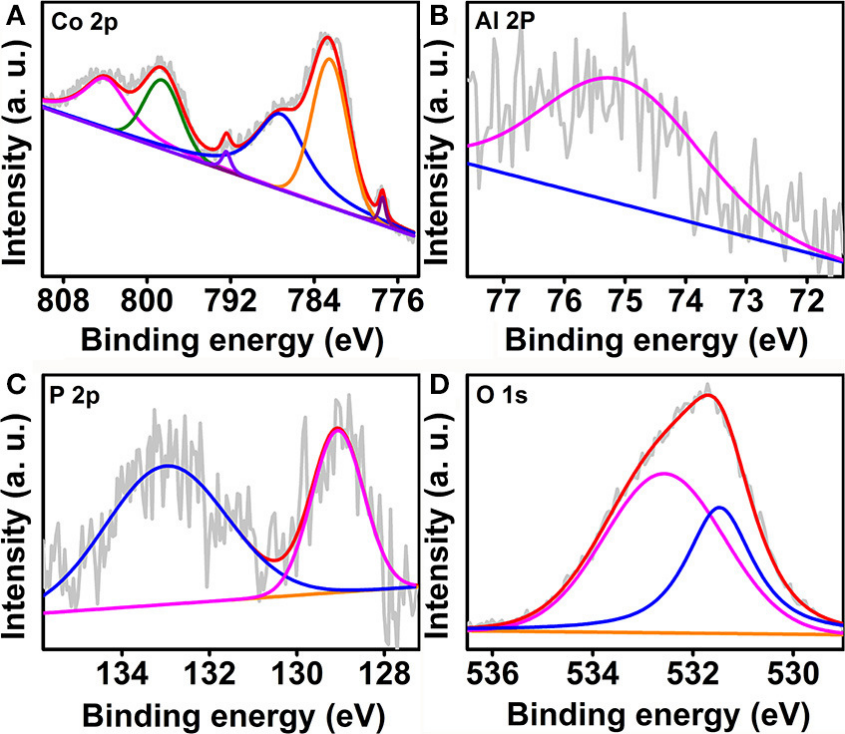
High resolution XPS spectra of **(A)** Co 2p, **(B)** Al 2p, **(C)** P 2p, and **(D)** O 1s regions for CoAl-LDH@Co-Al-P.

The obtained CoAl-LDH@Co-Al-P was also analyzed by x-ray photoelectron spectroscopy (XPS) to identify the elemental compositions and chemical states (Chen et al., 2018b). As shown in Figure 2A, the high-resolution Co 2p spectrum of CoAl-LDH@Co-Al-P has six peaks at 777.5, 782.5, 787.2, 792.5, 798.5, and 803.9 eV. The peaks located at 777.5 and 792.5 eV are assigned to Co 2p_3/2_ and Co 2p_1/2_ of metallic Co, respectively (Wei et al., 2015; Tian et al., 2019). Moreover, the peaks observed at 787.2 and 798.5 eV are attributed to Co 2p_3/2_ and Co 2p_1/2_, accompanying two satellite peaks centered at 787.2 and 803.9 eV, respectively (Yu et al., 2016). In the case of the Al 2p spectrum, a characteristic peak at 75.1 eV was observed and can be attributed to Al (III) (Figure 2B) (Zhang R. et al., 2017). Figure 2C shows the P 2p XPS spectrum of CoAl-LDH@Co-Al-P. One peak at 129.1 eV was attributable to the metal phosphide, and the other at 133.0 eV corresponded to the oxidized P species, such as phosphite or phosphate (P-O or PO_x_) (Li et al., 2016; Xu et al., 2020). The P 2p binding energy located at 129.1 eV was negatively shifted when compared with the one in CoP (Grosvenor et al., 2005), which implied that the P atoms were electronegative. The negatively charged P atoms not only serve as bases to attract hydrogen protons but also prompt H_2_ dissociation from the metal centers (Xiao et al., 2015; Shi and Zhang, 2016). From the high-resolution XPS spectrum of O 1s (Figure 2D), the peak that appeared at 531.5 eV was characteristic of the defect site of hypoxic coordination. Note that the peak at 532.6 eV was related to the adsorbed water molecule on the surface of the catalyst (Zhang P. et al., 2018; Zhang T. et al., 2018). All the aforementioned results confirm that the amorphous Co-Al-P layer was constructed on the surface of CoAl-LDH.

**Figure 3:**
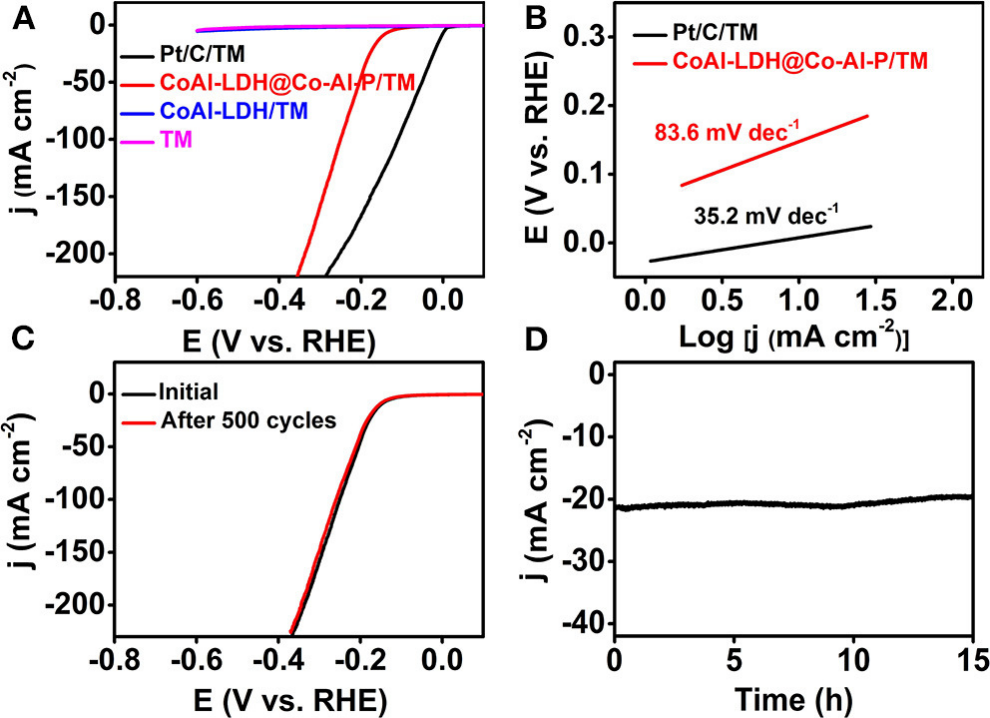
**(A)** HER polarization curves for Pt/C/TM, Ti mesh, CoAl-LDH/TM, and CoAl-LDH@Co-Al-P/TM. **(B)** The corresponding Tafel plots for Pt/C/TM and CoAl-LDH@Co-Al-P/TM. **(C)** LSV curves recorded before and after 500 cycles for CoAl-LDH@Co-Al-P/TM. **(D)** The time-dependent current density curve for CoAl-LDH@Co-Al-P/TM in 1 M PBS.

As a proof-of-concept application, the electrocatalytic HER performance of CoAl-LDH@Co-Al-P/TM was determined in PBS (1 M, pH = 7) using a standard three-electrode setup with a scan rate of 2 mV s^−1^. For comparison, the HER activity of bare Ti mesh, Pt/C on Ti mesh (Pt/C/TM), and CoAl-LDH/TM were also measured in the same conditions. All the polarization curves were corrected due to the effect of ohmic loss and all the potentials were calculated on a reversible hydrogen electrode (RHE) scale unless specifically noted. The characteristic polarization curves of the samples are shown in Figure 3A. As expected, Pt/C/TM exhibited very high energy efficiency for the HER. Bare Ti mesh and CoAl-LDH/TM were nearly inert to electrochemical water reduction in the measurement voltage window. Significantly, CoAl-LDH@Co-Al-P/TM was advantageous in the neutral HER, which only needed an overpotential of 150 mV to afford the catalytic current density of 10 mA cm^−2^. Furthermore, the cathodic current promptly increased when the potential became more negative. The lower overpotential represented the superior catalytic performance of CoAl-LDH@Co-Al-P/TM during hydrogen generation. Moreover, CoAl-LDH@Co-Al-P/TM acted as an efficient electrocatalyst comparable to or better than most of the reported Co-based HER catalysts in neutral solutions, as listed in Supplementary Table 1 in the electronic supplementary information. Furthermore, the reaction kinetics were estimated by Tafel slopes, which were obtained by fitting the linear sweep voltammetry (LSV) curves. Figure 3B depicts that the Tafel slopes of Pt/C/TM and CoAl-LDH@Co-Al-P/TM are 35.2 and 83.6 mV dec^−1^, respectively. A Tafel slope value of 83.6 mV dec^−1^ for CoAl-LDH@Co-Al-P/TM indicates that the Heyrovsky step serves as the rate-determining step and the HER process follows the Volmer-Heyrovsky mechanism (Liu et al., 2015). Therefore, we concluded that CoAl-LDH@Co-Al-P/TM has potential practical applications in gas-evolving neutral electrolytes.

Stability and durability were further used to evaluate the HER performance of the electrocatalyst. The stability of CoAl-LDH@Co-Al-P/TM was assessed via the cyclic voltammetry (CV) method in 1 M PBS. The catalytic activity of CoAl-LDH@Co-Al-P/TM exhibited negligible degradation compared with the initial one after 500 continuous CV scans with a scan rate of 100 mV s^−1^, indicating the excellent operational stability of the catalyst (Figure 3C). Simultaneously, long-term durability of CoAl-LDH@Co-Al-P/TM toward the HER is shown in Figure 3D. The catalyst maintained its catalytic performance for at least 15 h with only a slight activity loss in neutral electrolytes.

**Figure 4:**
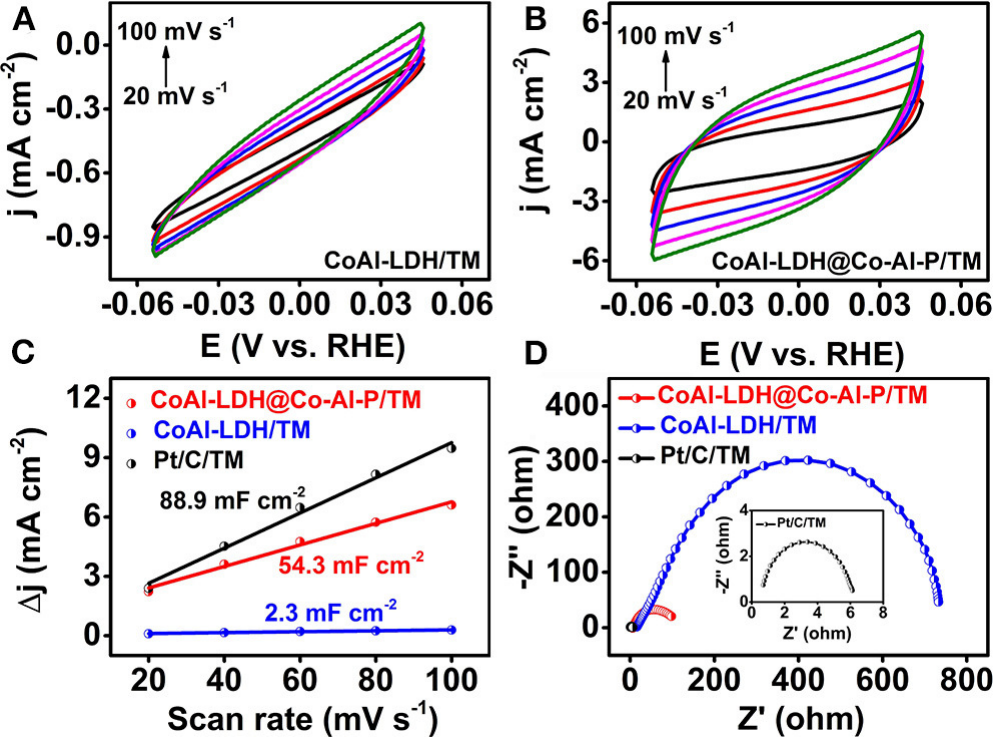
CVs of **(A)** CoAl-LDH/TM and **(B)** CoAl-LDH@Co-Al-P/TM at different scan rates increasing from 20 to 100 mV s^−1^ in 1 M PBS. **(C)** Estimation of ECSAs by CV. The cathodic and anodic current density difference when plotted against scan rate to calculate the Cdl value which was proportional to ECSA. **(D)** EIS data of Pt/C/TM, CoAl-LDH/TM, and CoAl-LDH@Co-Al-P/TM at the open-circuit potential (inset: EIS spectrum of Pt/C/TM).

The electrochemically active surface area (ECSA) was a key descriptor for the HER electrocatalyst and was evaluated by double-layer capacitance (C_dl_) in the non-faradic potential window (Figures 4A,B) (Liu et al., 2019; Zhang et al., 2019). As expected and shown in Figure 4C, the capacitance of CoAl-LDH@Co-Al-P/TM was measured to be 54.3 mF cm^−2^, which was lower than that of Pt/C/TM but remarkably larger than that of CoAl-LDH/TM (2.3 mF cm^−2^). This result represents a much higher electrochemically active surface area and the more advantageous catalytic active sites of CoAl-LDH@Co-Al-P/TM. Electrochemical impedance spectroscopy (EIS) analysis was also used to gain deep insights into the charge transfer characteristics of the electrocatalysts (Wang J. et al., 2017; Kim et al., 2019). It was clear that CoAl-LDH@Co-Al-P/TM had a smaller semicircular diameter than that of CoAl-LDH/TM, suggesting a lower impedance and a faster electron-transfer process of CoAl-LDH@Co-Al-P/TM (Figure 4D). Consequently, it was reasonably assumed that the enhanced performance in the HER might have originated from the intrinsic nature of CoAl-LDH@Co-Al-P/TM. Correspondingly, the Faradic efficiency of CoAl-LDH@Co-Al-P/TM was close to 100% which was obtained by comparing the amount of hydrogen experimentally generated with that of theoretically calculated amounts (Supplementary Figure 2) (Wang et al., 2018b; Chen et al., 2020c). Taken together, CoAl-LDH@Co-Al-P/TM was demonstrated to be an active and stable catalyst toward the HER at a neutral pH.

## Conclusions

In summary, a core-shell CoAl-LDH@Co-Al-P/TM nanosheet array had been successfully constructed via a *in-situ* wet phosphidation strategy. CoAl-LDH@Co-Al-P/TM was an efficient HER catalyst that only demanded an overpotential of 150 mV to acquire a catalytic current density of 10 mA cm^−2^ at a neutral pH, and exhibited good electrochemical stability for long-term operation. This work not only provides us with an effective and stable earth-abundant catalyst toward the HER in neutral conditions, but offers us a green and facile strategy to the synthesis of amorphous CoAl-based electrocatalysts with a nanoarray feature.

## Experimental Section

### Fabrication of CoAl-LDH@Co-Al-P/TM

In a typical synthesis process, the CoAl layered double hydroxide (CoAl-LDH) was prepared via a hydrothermal reaction. In brief, 1.75 g of Co(NO_3_)_2_·6H_2_O, 0.23 g of Al(NO_3_)_3_·9H_2_O, 1.20 g of CO(NH_2_)_2_, and 0.30 g of NH_4_F were dissolved in 40 mL of deionized water. After vigorous stirring for 30 min, the obtained solution was poured into a 50 mL Teflon-lined stainless steel autoclave and a piece of Ti mesh (2 cm × 3 cm) was put into the solution. Then the autoclave was sealed and kept at 110°C for 8 h in an electric oven. After naturally cooling down to room temperature, CoAl-LDH grown on Ti mesh (CoAl-LDH/TM) was obtained and alternately washed with ultrapure water and ethanol several times. Finally, CoAl-LDH/TM was vacuum dried at 60°C for 6 h.

CoAl-LDH/TM, a Pt wire, and a saturated calomel electrode (SCE) were used as the working electrode, counter electrode, and reference electrode in a typical three-electrode system, respectively. A constant potential of −1.2 V vs. SCE was applied for 6,400 s in a specific solution, which contained 3 g of Na_3_C_6_H_5_O_7_·2H_2_O, 3 g of (NH_4_)_2_SO_4_, and 3 g of NaH_2_PO_2_·H_2_O in 50 mL of water.

### Characterization

The XRD pattern was acquired on a LabX XRD-6100 x-ray diffractometer with Cu Kα radiation (40 kV, 30 mA) with a wavelength of 0.154 nm (SHIMADZU, Japan). The x-ray photoelectron spectroscopy measurements were performed on an ESCALABMK II X-ray photoelectron spectrometer using Mg as the exciting source. The scanning electron microscopy images were collected on an S-4800 field emission scanning electron microscope at an accelerating voltage of 20 kV (HITACHI, Japan). The transmission electron microscopy images were obtained on a Zeiss Libra 200FE transmission electron microscope operated at 200 kV.

### Electrochemical Measurement

The electrochemical data were recorded using a CHI 660E electrochemical workstation (Chenhua, Shanghai) in a three-electrode system. CoAl-LDH@Co-Al-P/TM, a graphite plate, and a saturated calomel electrode were used as the working electrode, counter electrode, and reference electrode, respectively. The potentials were corrected against an IR drop and calibrated to the reversible hydrogen electrode (RHE): E (RHE) = E (SCE) + (0.242+ 0.059 pH) = E (SCE) + 0.655 V. All the tests were performed at 298 K in 1 M PBS solution.

## Data Availability Statement

The raw data supporting the conclusions of this article will be made available by the authors, without undue reservation.

## Author Contributions

WT and ZS performed the experimental work. ZS and JX wrote the manuscript. XZ performed the characterization of related materials. ZW and BT revised the manuscript. All authors contributed to the article and approved the submitted version.

## Conflict of Interest

The authors declare that the research was conducted in the absence of any commercial or financial relationships that could be construed as a potential conflict of interest.

## References

[B1] Chen L. Sun B. Wang X. Qiao F. Ai S. (2013). 2D ultrathin nanosheets of Co-Al layered double hydroxides prepared in _L_-asparagine solution: enhanced peroxidase-like activity and colorimetric detection of glucose. J. Mater. Chem. B 1:2268. 10.1039/c3tb00044c 32260880

[B2] Chen Y. Yu J. Jia J. Liu F. Zhang Y. Xiong G. (2020b). Metallic Ni_3_Mo_3_N porous microrods with abundant catalytic sites as efficient electrocatalyst for large current density and superstability of hydrogen evolution reaction and water splitting. Appl. Catal. B Environ. 272:118956 10.1016/j.apcatb.2020.118956

[B3] Chen Z. Fei B. Hou M. Yan X. Chen M. Qing H. (2020a). Ultrathin prussian blue analogue nanosheet arrays with open bimetal centers for efficient overall water splitting. Nano Energy 68:104371 10.1016/j.nanoen.2019.104371

[B4] Chen Z. Ha Y. Jia H. Yan X. Chen M. Liu M. (2019). Oriented transformation of Co-LDH into 2D/3D ZIF-67 to achieve Co-N-C hybrids for efficient overall water splitting. Adv. Energy Mater. 9:1803918 10.1002/aenm.201803918

[B5] Chen Z. Qing H. Zhou K. Sun D. Wu R. (2020c). Metal-organic framework-derived nanocomposites for electrocatalytic hydrogen evolution reaction. Prog. Mater. Sci. 108:100618 10.1016/j.pmatsci.2019.100618

[B6] Chen Z. Song Y. Cai J. Zheng X. Han D. Wu Y.. (2018a). Tailoring the d-band centers enables Co_4_N nanosheets to be highly active for hydrogen evolution catalysis. Angew. Chem. Int. Ed. 57, 5076–5080. 10.1002/anie.201801834 29498161

[B7] Chen Z. Wu R. Liu Y. Ha Y. Guo Y. Sun D.. (2018b). Ultrafine Co nanoparticles encapsulated in carbon-nanotubes-grafted graphene sheets as advanced electrocatalysts for the hydrogen evolution reaction. Adv. Mater 30:1802011. 10.1002/adma.201802011 29888482

[B8] Cheng J. P. Fang J. H. Li M. Zhang W. F. Liu F. Zhang X. B. (2013). Enhanced electrochemical performance of CoAl-layered double hydroxide nanosheet arrays coated by platinum films. Electrochim. Acta 114:68–75. 10.1016/j.electacta.2013.10.029

[B9] Chi J.-Q. Gao W.-K. Lin J.-H. Dong B. Yan K.-L. Qin J.-F. (2019). N, P dual-doped hollow carbon spheres supported MoS_2_ hybrid electrocatalyst for enhanced hydrogen evolution reaction. Catal. Today 330, 259–267. 10.1016/j.cattod.2018.03.003

[B10] Chow J. Kopp R. J. Portney P. R. (2003). Energy resources and global development. Science 302, 1528–1531. 10.1126/science.1091939 14645838

[B11] Chu S. Majumdar A. (2012). Opportunities and challenges for a sustainable energy future. Nature 488, 294–303. 10.1038/nature11475 22895334

[B12] Faber M. S. Dziedzic R. Lukowski M. A. Kaiser N. S. Ding Q. Jin S. (2014). High-performance electrocatalysis using metallic cobalt pyrite (CoS_2_) micro- and nanostructures. J. Am. Chem. Soc. 136, 10053–10061. 10.1021/ja504099w 24901378

[B13] Fei B. Chen Z. Ha Y. Wang R. Yang H. Xu H. (2020). Anion-cation co-substitution activation of spinel CoMoO_4_ for efficient oxygen evolution reaction. Chem. Eng. J. 394:124926 10.1016/j.cej.2020.124926

[B14] Grosvenor A. P. Wik S. D. Cavell R. G. Mar A. (2005). Examination of the bonding in binary transition-metal monophosphides MP (M = Cr, Mn, Fe, Co) by X-Ray photoelectron spectroscopy. Inorg. Chem. 44, 8988–8998. 10.1021/ic051004d 16296854

[B15] Hu J. Cao X. Zhao X. Chen W. Lu G. Dan Y.. (2019). Catalytically active sites on Ni_5_P_4_ for efficient hydrogen evolution reaction from atomic scale calculation. Front. Chem. 7:444. 10.3389/fchem.2019.00444 31263695 PMC6590065

[B16] Hu J. Huang B. Zhang C. Wang Z. An Y. Zhou D. (2017). Engineering stepped edge surface structures of MoS_2_ sheet stacks to accelerate the hydrogen evolution reaction. Energy Environ. Sci. 10, 593–603. 10.1039/C6EE03629E

[B17] Huang L. Zou Y. Chen D. Wang S. (2019). Electronic structure regulation on layered double hydroxides for oxygen evolution reaction. Chin. J. Catal. 40, 1822–1840. 10.1016/S1872-2067(19)63284-5

[B18] Huang T. Shen T. Gong M. Deng S. Lai C. Liu X. (2019). Ultrafine Ni-B nanoparticles for efficient hydrogen evolution reaction. Chin. J. Catal. 40, 1867–1873. 10.1016/S1872-2067(19)63331-0

[B19] Kim M. Anjum M. A. R. Lee M. Lee B. J. Lee J. S. (2019). Activating MoS_2_ basal plane with Ni_2_P nanoparticles for Pt-like hydrogen evolution reaction in acidic media. Adv. Funct. Mater. 29:1809151 10.1002/adfm.201809151

[B20] Li D. Baydoun H. Verani C. N. Brock S. L. (2016). Efficient water oxidation using CoMnP nanoparticles. J. Am. Chem. Soc. 138, 4006–4009. 10.1021/jacs.6b01543 26972408

[B21] Li N. Liu X. Li G.-D. Wu Y. Gao R. Zou X. (2017). Vertically grown CoS nanosheets on carbon cloth as efficient hydrogen evolution electrocatalysts. Int. J. Hydrogen Energy 42, 9914–9921. 10.1016/j.ijhydene.2017.01.191

[B22] Li R. Zhou D. Luo J. Xu W. Li J. Li S. (2017). The urchin-like sphere arrays Co_3_O_4_ as a bifunctional catalyst for hydrogen evolution reaction and oxygen evolution reaction. J. Power Sources 341, 250–256. 10.1016/j.jpowsour.2016.10.096

[B23] Ling T. Yan D. Y. Wang H. Jiao Y. Hu Z. Zheng Y.. (2017). Activating cobalt(II) oxide nanorods for efficient electrocatalysis by strain engineering. Nat. Commun. 8:1509. 10.1038/s41467-017-01872-y 29138406 PMC5686154

[B24] Liu Q. Tian J. Cui W. Jiang P. Cheng N. Asiri A. M.. (2014). Carbon nanotubes decorated with CoP nanocrystals: a highly active non-noble-metal nanohybrid electrocatalyst for hydrogen evolution. Angew. Chem. Int. Ed. 53, 6710–6714. 10.1002/anie.201404161 24845625

[B25] Liu T. Li P. Yao N. Cheng G. Chen S. Luo W.. (2019). CoP-doped MOF-based electrocatalyst for pH-universal hydrogen evolution reaction. Angew. Chem. Int. Ed. 58, 4679–4684. 10.1002/anie.201901409 30716195

[B26] Liu T. Liu Q. Asiri A. M. Luo Y. Sun X. (2015). An amorphous CoSe film behaves as an active and stable full water-splitting electrocatalyst under strongly alkaline conditions. Chem. Commun. 51, 16683–16686. 10.1039/C5CC06892D 26431349

[B27] Shi Y. Zhang B. (2016). Recent advances in transition metal phosphide nanomaterials: synthesis and applications in hydrogen evolution reaction. Chem. Soc. Rev. 45, 1529–1541. 10.1039/C5CS00434A 26806563

[B28] Sun Z. Zhang J. Xie J. Zheng X. Wang M. Li X. (2018). High-performance alkaline hydrogen evolution electrocatalyzed by a Ni_3_N-CeO_2_ nanohybrid. Inorg. Chem. Front. 5, 3042–3045. 10.1039/C8QI00905H

[B29] Tian H. Liu X. Dong L. Ren X. Liu H. Price C. A. H.. (2019). Enhanced hydrogenation performance over hollow structured Co-CoO_x_@N-C capsules. Adv. Sci. 6:1900807. 10.1002/advs.201900807 31763134 PMC6865004

[B30] Tian J. Liu Q. Asiri A. M. Sun X. (2014). Self-supported nanoporous cobalt phosphide nanowire arrays: an efficient 3D hydrogen-evolving cathode over the wide range of pH 0-14. J. Am. Chem. Soc. 136, 7587–7590. 10.1021/ja503372r 24830333

[B31] Wang C. J. Wu Y. A. Jacobs R. M. J. Warner J. H. Williams G. R. O'Hare D. (2011). Reverse micelle synthesis of Co-Al LDHs: control of particle size and magnetic properties. Chem. Mater. 23, 171–180. 10.1021/cm1024603

[B32] Wang J. Chen J. W. Chen J. D. Zhu H. Zhang M. Du M. L. (2017). Designed synthesis of size-controlled Pt-Cu alloy nanoparticles encapsulated in carbon nanofibers and their high efficient electrocatalytic activity toward hydrogen evolution reaction. Adv. Mater. Interfaces 4:1700005 10.1002/admi.201700005

[B33] Wang J. Cui W. Liu Q. Xing Z. Asiri A. M. Sun X. (2016). Recent progress in cobalt-based heterogeneous catalysts for electrochemical water splitting. Adv. Mater 28:215–230. 10.1002/adma.201502696 26551487

[B34] Wang Y. Zhang B. Pan W. Ma H. Zhang J. (2017). 3 D porous nickel-cobalt nitrides supported on nickel foam as efficient electrocatalysts for overall water splitting. ChemSusChem 10, 4170–4177. 10.1002/cssc.201701456 28857449

[B35] Wang Z. Ren X. Luo Y. Wang L. Cui G. Xie F.. (2018a). An ultrafine platinum-cobalt alloy decorated cobalt nanowire array with superb activity toward alkaline hydrogen evolution. Nanoscale 10, 12302–12307. 10.1039/C8NR02071J 29932199

[B36] Wang Z. Ren X. Shi X. Asiri A. M. Wang L. Li X. (2018b). A platinum oxide decorated amorphous cobalt oxide hydroxide nanosheet array towards alkaline hydrogen evolution. J. Mater. Chem. A 6, 3864–3868. 10.1039/C8TA00241J

[B37] Wei Z. Wang J. Mao S. Su D. Jin H. Wang Y. (2015). *In situ*-generated Co^0^-Co_3_O_4_/N-doped carbon nanotubes hybrids as efficient and chemoselective catalysts for hydrogenation of nitroarenes. ACS Catal. 5, 4783–4789. 10.1021/acscatal.5b00737

[B38] Xiao P. Chen W. Wang X. (2015). A review of phosphide-based materials for electrocatalytic hydrogen evolution. Adv. Energy Mater. 5:1500985 10.1002/aenm.201500985

[B39] Xie J. Xie Y. (2015). Structural engineering of electrocatalysts for the hydrogen evolution reaction: order or disorder? ChemCatChem 7, 2568–2580. 10.1002/cctc.201500396

[B40] Xu H. Jia H. Fei B. Ha Y. Li H. Guo Y. (2020). Charge transfer engineering via multiple heteroatom doping in dual carbon-coupled cobalt phosphides for highly efficient overall water splitting. Appl. Catal. B 268:118404 10.1016/j.apcatb.2019.118404

[B41] Xu K. Ding H. Zhang M. Chen M. Hao Z. Zhang L.. (2017). Regulating water-reduction kinetics in cobalt phosphide for enhancing HER catalytic activity in alkaline solution. Adv. Mater. 29:1606980. 10.1002/adma.201606980 28513886

[B42] Xue S. Zhang W. Zhang Q. Du J. Cheng H.-M. Ren W. (2020). Heterostructured Ni-Mo-N nanoparticles decorated on reduced graphene oxide as efficient and robust electrocatalyst for hydrogen evolution reaction. Carbon 165, 122–128. 10.1016/j.carbon.2020.04.066

[B43] Yu L. Shi N. Liu Q. Wang J. Yang B. Wang B. (2014) Facile synthesis of exfoliated Co-Al LDH-carbon nanotube composites with high performance as supercapacitor electrodes. Phys. Chem. Chem. Phys. 16, 17936–17942. 10.1039/c4cp02020k 25050421

[B44] Yu X. Zhang S. Li C. Zhu C. Chen Y. Gao P.. (2016). Hollow CoP nanopaticle/N-doped graphene hybrids as highly active and stable bifunctional catalysts for full water splitting. Nanoscale 8, 10902–10907. 10.1039/C6NR01867J 27181021

[B45] Zai J. Liu Y. Li X. Ma Z. Qi R. Qian X. (2017). 3D hierarchical Co-Al layered double hydroxides with long-term stabilities and high rate performances in supercapacitors. Nano Micro Lett. 9:21. 10.1007/s40820-016-0121-5 30460317 PMC6223799

[B46] Zhang A. Wang C. Xu Q. Liu H. Wang Y. Xia Y. (2015). A hybrid aerogel of Co-Al layered double hydroxide/graphene with three-dimensional porous structure as a novel electrode material for supercapacitors. RSC Adv. 5, 26017–26026. 10.1039/C5RA00103J

[B47] Zhang C. Huang Y. Yu Y. Zhang J. Zhuo S. Zhang B. (2017). Sub-1.1 nm ultrathin porous CoP nanosheets with dominant reactive {200} facets: a high mass activity and efficient electrocatalyst for the hydrogen evolution reaction. Chem. Sci. 8, 2769–2775. 10.1039/C6SC05687C 28553512 PMC5426437

[B48] Zhang G. Wang B. Bi J. Fang D. Yang S. (2019) Constructing ultrathin CoP nanomeshes by Er-doping for highly efficient bifunctional electrocatalyst for overall water splitting. J. Mater. Chem. A 7, 5769–5778. 10.1039/c9ta00530g

[B49] Zhang H. Li Y. Zhang G. Xu T. Wan P. Sun X. (2015). A metallic CoS_2_ nanopyramid array grown on 3D carbon fiber paper as an excellent electrocatalyst for hydrogen evolution. J. Mater. Chem. A 3, 6306–6310. 10.1039/C5TA00707K

[B50] Zhang P. Xu B. Chen G. Gao C. Gao M. (2018). Large-scale synthesis of nitrogen doped MoS_2_ quantum dots for efficient hydrogen evolution reaction. Electrochim. Acta 270, 256–263. 10.1016/j.electacta.2018.03.097

[B51] Zhang R. Tang C. Kong R. Du G. Asiri A. M. Chen L.. (2017). Al-doped CoP nanoarray: a durable water-splitting electrocatalyst with superhigh activity. Nanoscale 9, 4793–4800. 10.1039/C7NR00740J 28349153

[B52] Zhang T. Wu M.-Y. Yan D.-Y. Mao J. Liu H. Hu W.-B.. (2018). Engineering oxygen vacancy on NiO nanorod arrays for alkaline hydrogen evolution. Nano Energy 43, 103–109. 10.1016/j.nanoen.2017.11.01529741363

[B53] Zhou W. Jia J. Lu J. Yang L. Hou D. Li G. (2016). Recent developments of carbon-based electrocatalysts for hydrogen evolution reaction. Nano Energy 28, 29–43. 10.1016/j.nanoen.2016.08.027

[B54] Zhu J. Hu L. Zhao P. Lee L. Y. S. Wong K.-Y. (2020). Recent advances in electrocatalytic hydrogen evolution using nanoparticles. Chem. Rev. 120, 851–918. 10.1021/acs.chemrev.9b00248 31657904

