# Supplementary material for: In-situ Formation of Amorphous Co-Al-P Layer on CoAl Layered Double Hydroxide Nanoarray as Neutral Electrocatalysts for Hydrogen Evolution Reaction
Source: Front Chem. 2020 Oct 22;8:552795. doi: 10.3389/fchem.2020.552795 (PMC7642338; doi:10.3389/fchem.2020.552795)
Supplement: Supplementary file 1 [file Data_Sheet_1.pdf]

Electronic Supplementary Information

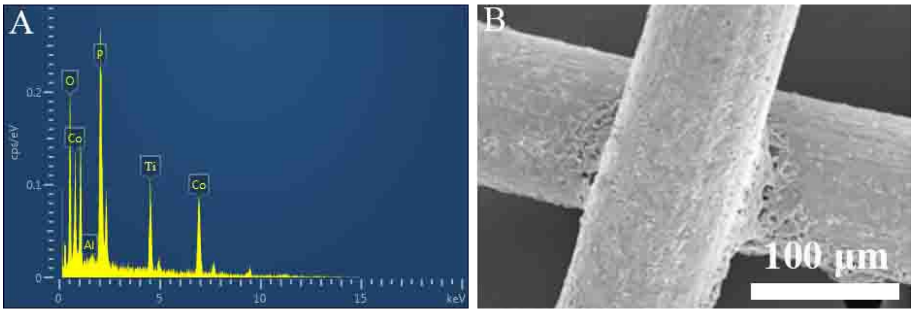

FIGURE S1| (A) EDX spectrum and **(B)** SEM image for CoAl-LDH@Co-Al-P/TM.

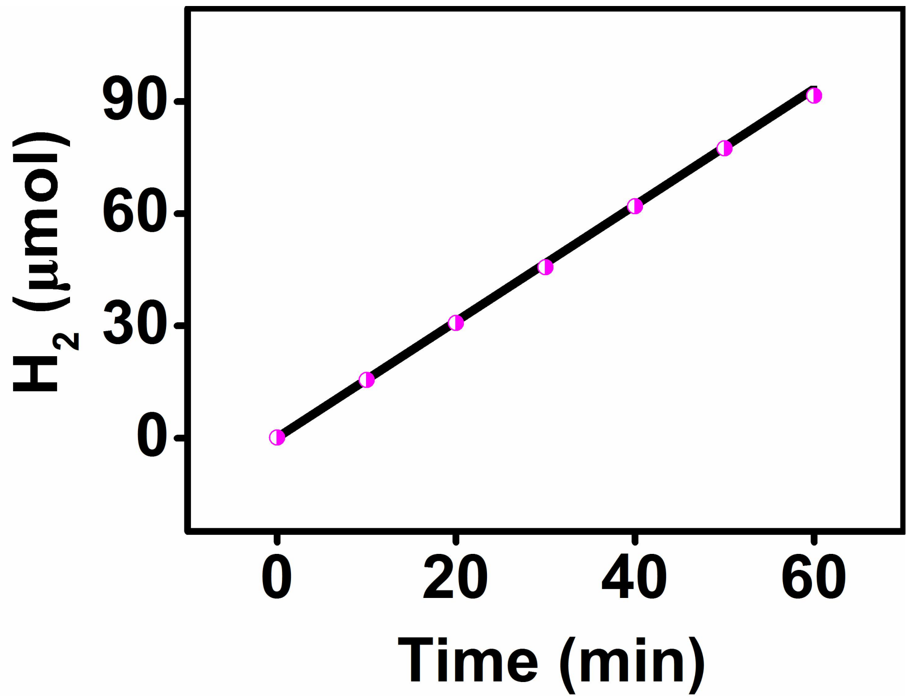

FIGURE S2| Experimentally generated (dot) and theoretically calculated (line) hydrogen amount versus time for CoAl-LDH@Co-Al-P/TM in 1 M PBS.

Table S1. Comparison of HER performance for CoAl-LDH@Co-Al-P/TM with other non-noble-metal electrocatalysts in 1 M PBS.

Catalyst	j (mA cm^{-2})	η (mV)	Electrolyte	Ref.
CoAl-LDH@Co-Al-P/TM	10	150	1 M PBS	This work
Co-NCNT/CC	10	170	1 M PBS	1
Co-S film	10	~165	1 M PBS	2
Co_2N/TM	10	290	1 M PBS	3
FeP/Ti	10	200	1 M PBS	4
$FeMoS_4$ NRA/CC	10	204	1 M PBS	5
WP NAs/CC	10	200	1 M PBS	6
FeP NAs/CC	10	202	1 M PBS	7
np-CoP NWs/Ti	10	178	1 M PBS	8
S-$NiFe_2O_4$/NF	10	197	1 M PBS	9
Co-$Mo_{0.4}$-S	10	213	1 M PBS	10
SiO_2/PPy NTs-CFs	10	~185	1 M PBS	11
Cu PDA-3	10	290	1 M PBS	12
Ni_2P@NPCNFs	10	185.3	1 M PBS	13
Mn-FeP	10	157	1 M PBS	14
WS_2/CoS_2/CC	10	175	1 M PBS	15
CNT@NPC-900	10	440	1 M PBS	16
$MnMoO_4$ NSA/NF	10	161	1 M PBS	17

References

1 Xing Z., Liu Q., Xing W., Asiri A. M. and Sun X. (2015) Interconnected Co-entrapped, N-doped carbon nanotube film as active hydrogen evolution cathode over the whole pH range. *ChemSusChem* 8: 1850-1855. doi:10.1002/cssc.201500138.

2 Sun Y., Liu C., Grauer D. C., Yano J., Long J. R., Yang P. and Chang C. J. (2013) Electrodeposited cobalt-sulfide catalyst for electrochemical and photoelectrochemical hydrogen generation from water. *J Am Chem Soc* 135: 17699-17702. doi:10.1021/ja4094764.

3 Zhang L., Xie L., Ma M., Qu F., Du G., Asiri A. M., Chen L. and Sun X. (2017) Co-based nanowire films as complementary hydrogen- and oxygen-evolving electrocatalysts in neutral electrolyte. *Catal Sci Technol* 7: 2689-2694. doi:10.1039/C7CY00703E.

4 Pu Z., Tang C. and Luo Y. (2015) Ferric phosphide nanoparticles film supported on titanium plate: A high-performance hydrogen evolution cathode in both acidic and neutral solutions. *Int J Hydrogen Energy* 40: 5092-5098. doi:10.1016/j.ijhydene.2015.02.026.

5 Ren X., Wang W., Ge R., Hao S., Qu F., Du G., Asiri A. M., Wei Q., Chen L. and Sun X. (2017) An amorphous $FeMoS_4$ nanorod array toward efficient hydrogen evolution electrocatalysis under neutral conditions. *Chem Commun* 53: 9000-9003. doi:10.1039/C7CC03702C.

6 Pu Z., Liu Q., Asiri A. M. and Sun X. (2014) Tungsten phosphide nanorod arrays directly grown on carbon cloth: A highly efficient and stable hydrogen evolution cathode at all pH values. *ACS Appl Mater Interfaces* 6: 21874-21879. doi:10.1021/am5060178.

7 Liang Y., Liu Q., Asiri A. M., Sun X. and Luo Y. (2014) Self-supported FeP nanorod arrays: A cost-effective 3D hydrogen evolution cathode with high catalytic activity. *ACS Catal* 4: 4065-4069. doi:10.1021/cs501106g.

8 Gu S., Du H., Asiri A. M., Sun X. and Li C. M. (2014) Three-dimensional interconnected network of nanoporous CoP nanowires as an efficient hydrogen evolution cathode. *Phys Chem Chem Phys* 16: 16909-16913. doi:10.1039/C4CP02613F.

9 Liu J., Zhu D., Ling T., Vasileff A. and Qiao S.-Z. (2017) S-$NiFe_2O_4$ ultra-small nanoparticle built nanosheets for efficient water splitting in alkaline and neutral pH. *Nano Energy* 40: 264-273. doi:10.1016/j.nanoen.2017.08.031.

10 Zhou L., Han Z., Li W., Leng W., Yu Z. and Zhao Z. (2020) Hierarchical Co-Mo-S nanoflowers as efficient electrocatalyst for hydrogen evolution reaction in neutral media. *J Alloy Compd* 844: 156108. doi:10.1016/j.jallcom.2020.156108.

11 Feng J.-X., Xu H., Ye S.-H., Ouyang G., Tong Y.-X. and Li G.-R. (2017) Silica-polypyrrole hybrids as high-performance metal-free electrocatalysts for the hydrogen evolution reaction in neutral media. *Angew Chem Int Ed* 56, 8120-8124. doi: 10.1002/anie.201702934.

12 Muthukumar P., Moon D. and Anthony S. P. (2019) Copper coordination polymer electrocatalyst for strong hydrogen evolution reaction activity in neutral medium: influence of coordination environment and network structure. *Catal Sci Technol* 9: 4347-4354. doi:10.1039/c9cy00759h.

13 Wang M.-Q., Ye C., Liu H., Xu M. and Bao S.-J. (2018) Nanosized metal phosphides embedded in nitrogen-doped porous carbon nanofibers for enhanced hydrogen evolution at all pH values. *Angew Chem Int Ed* 57:1963-1967. doi:10.1002/anie.201710150.

14 Wang M., Tuo Y., Li X., Hua Q., Du F. and Jiang L. (2019) Mesoporous Mn-doped FeP: facile synthesis and enhanced electrocatalytic activity for hydrogen evolution in a wide pH range. *ACS Sustainable Chem Eng* 7:12419-12427. doi:10.1021/acssuschemeng.9b01952.

15 Wu J., Chen T., Zhu C., Du J., Huang L., Yan J., Cai D., Guan C. and Pan C. (2020) Rational construction of a WS_2/CoS_2 heterostructure electrocatalyst for efficient hydrogen evolution at all pH values. *ACS Sustainable Chem Eng* 8: 4474-4480. doi:10.1021/acssuschemeng.9b07507.

16 Xiao F., Chen Z., Wu H., Wang Y., Cao E., Lu X., Wu Y. and Ren Z. (2019) Phytic acid-guided ultra-thin N,P co-doped carbon coated carbon nanotubes for efficient all-pH electrocatalytic hydrogen evolution. *Nanoscale* 11: 23027-23034. doi:10.1039/c9nr07362k.

17 Wen L., Sun Y., Zhang T., Bai Y., Li X., Lyu X., Cai W. and Li Y. (2018) $MnMoO_4$ nanosheet array: an efficient electrocatalyst for hydrogen evolution reaction with enhanced activity over a wide pH range. *Nanotechnology* 29: 335403. doi:10.1088/1361-6528/aac851.
